# Supplementary material for: Type 2 diabetes and its genetic susceptibility are associated with increased severity and mortality of COVID-19 in UK Biobank
Source: Commun Biol. 2024 Jan 24;7:122. doi: 10.1038/s42003-024-05799-1 (PMC10808197; doi:10.1038/s42003-024-05799-1)
Supplement: Supplementary file 2 — Supplementary Materials [file 42003_2024_5799_MOESM2_ESM.pdf]

Supplementary Materials to
Type 2 Diabetes and its Genetic Susceptibility are Associated with Increased Severity and Mortality of COVID-19 in UK Biobank

Aeyeon Lee[1], Jieun Seo[1], Seunghwan Park[1,2], Youngkwang Cho[1], Gaeun Kim[1], Jun Li[3], Liming Liang[4,5,#], Taesung Park[6,#] and Wonil Chung[1,5,#]

Supplementary Notes

Supplementary Note 1. Heritability and genetic correlations

We estimated the heritability and genetic correlation for various traits, including T2D, Body Mass Index (BMI), Waist-Hip Ratio (WHR), Coronary Heart Disease (CHD), Cardiovascular Disease (CVD), stroke, Diastolic Blood Pressure (DBP), Systolic Blood Pressure (SBP), Asthma Immune Disease, SARS-CoV-2 infection, and severe COVID-19 (Supplementary Table 1). Specifically, the heritability for obesity-related traits was 0.211 (SE=0.007) for BMI and 0.145 (SE=0.005) for WHR. The genetic correlation with T2D was 0.374 (SE=0.024) for WHR and 0.073 (SE=0.024) for BMI. Similarly, the genetic correlation with severe COVID-19 was 0.638 (SE=0.112) for BMI and 0.602 (SE=0.113) for WHR. This prompted us to explore the relationship between obesity-related traits and severe COVID-19.

Supplementary Note 2. Association of SARS-CoV-2 infection with T2D using logistic regression

To estimate the effect size of T2D and genetic susceptibility for T2D on SARS-CoV-2 infection, we conducted multivariable logistic regression models. T2D or T2D PRS was included in the logistic regression as an explanatory variable separately: $logit(P(Y_i = 1)) = \beta_0 + \beta_1 X_i + \boldsymbol{\beta_2 C_i}$ where Y_i is a binary outcome for SARS-CoV-2 infection (1: SARS-CoV-2 infection, 0: control) of *i*th individual; X_i is a T2D or T2D PRS; $\boldsymbol{C_i}$ is a set of clinical covariates including age, gender, vaccination status, BMI, genotyping array, and the top four PCs. The exp(β_1) is the odds ratio (OR) of T2D or T2D PRS, measuring the effect size on SARS-CoV-2 infection with T2D or a one-unit increase (=standard deviation [SD]) in T2D PRS. We also classified participants into three groups according to their categories of genetic risk (i.e., low, medium, and high genetic risk) based on PRS scores, and then included an indicator for these three PRS groups as a covariate in the model.

The estimated odds ratio (OR) for T2D was 1.033 (95% CI=1.004-1.062), indicating that the odds of SARS-CoV-2 infection among T2D patients were 1.033 times higher than controls (Supplementary Table 6). We also included T2D PRS as a covariate in the model and the estimated OR for T2D PRS was 1.012 (95% CI=1.002-1.023), meaning that the odds of SARS-CoV-2 infection among individuals with a one-unit increase (=SD) in T2D PRS were 1.012 times greater than those of the controls (Supplementary Table 6). We classified all participants into low, medium, and high PRS groups based on their PRS(T2D), and included the PRS group as a covariate (with low as the reference) in the model. The estimated OR for the high PRS group was significant (OR=1.032, 95% CI=1.000-1.066), meaning that the odds of SARS-CoV-2 infection in the high PRS group were 1.032 times greater than those in the low PRS group (Supplementary Table 6). Individuals with relatively high genetic susceptibility for T2D were more likely to be infected with SARS-CoV-2. In addition, we displayed the results of the analyses for the association of T2D-related diseases and their genetic susceptibilities with SARS-CoV-2 infection in Supplementary Table 7.

Supplementary Figures

Supplementary Figure 1. Manhattan Plots for T2D (N = 459,119, #Case: 37,110) (top), SARS-CoV-2 infection (N = 459,119, #Case: 101,271) (middle) and severe COVID-19 (N = 459,119, #Case:7,478) (bottom).

(a)

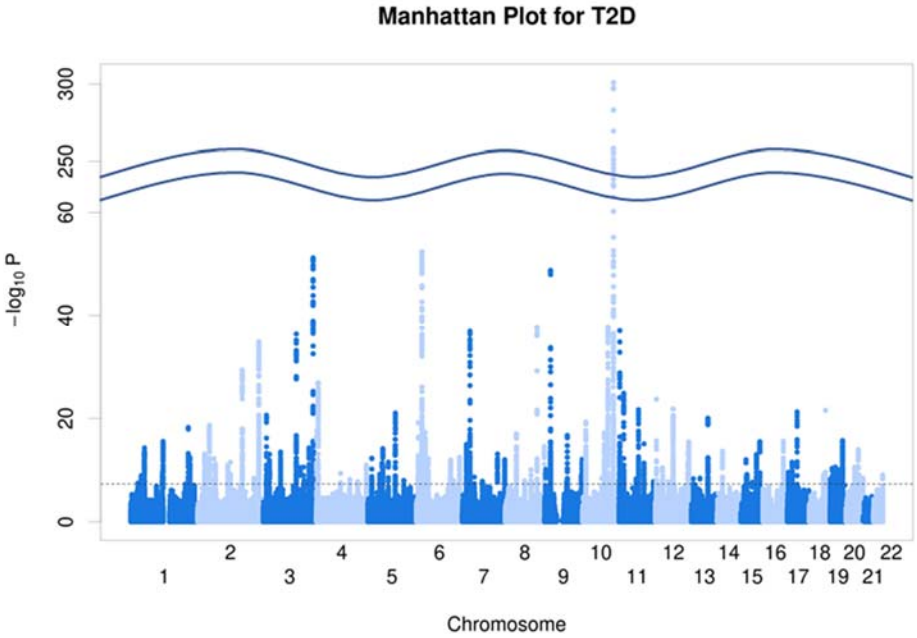

(b)

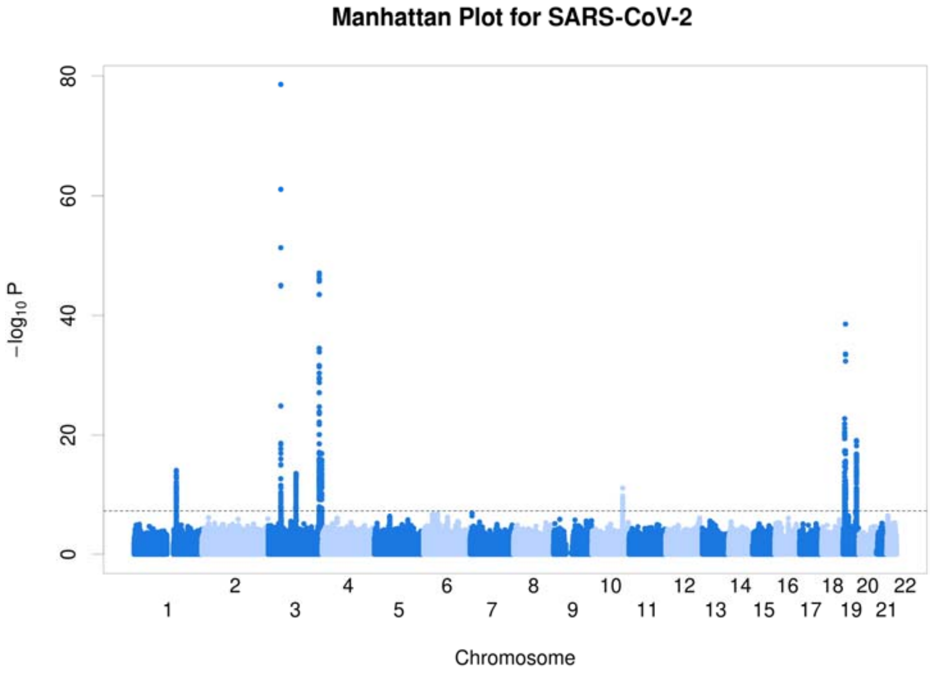

(c)

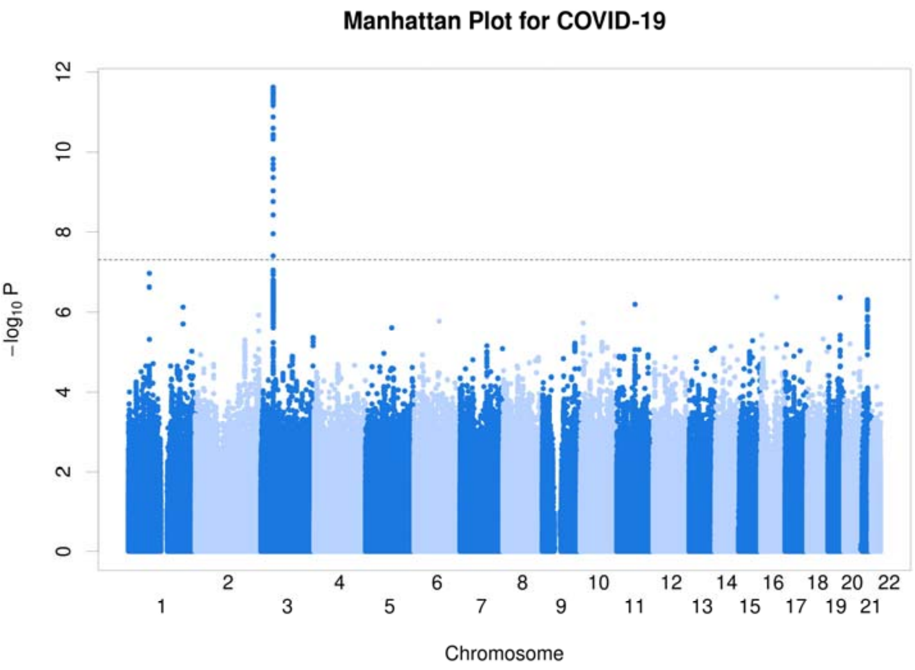
Manhattan Plot for COVID-19
-log10 P
0
2
4
6
8
10
12
Chromosome
1
2
3
4
5
6
7
8
9
10
11
12
13
14
15
16
17
18
19
20
21
22

Supplementary Figure 2. The ROC curves representing the prediction accuracy of PRS for T2D (top left) and adjusted PRS for T2D (top right), and plots for distribution of PRS stratified by T2D cases and controls (bottom left) and adjusted PRS stratified with T2D cases and controls (bottom right).

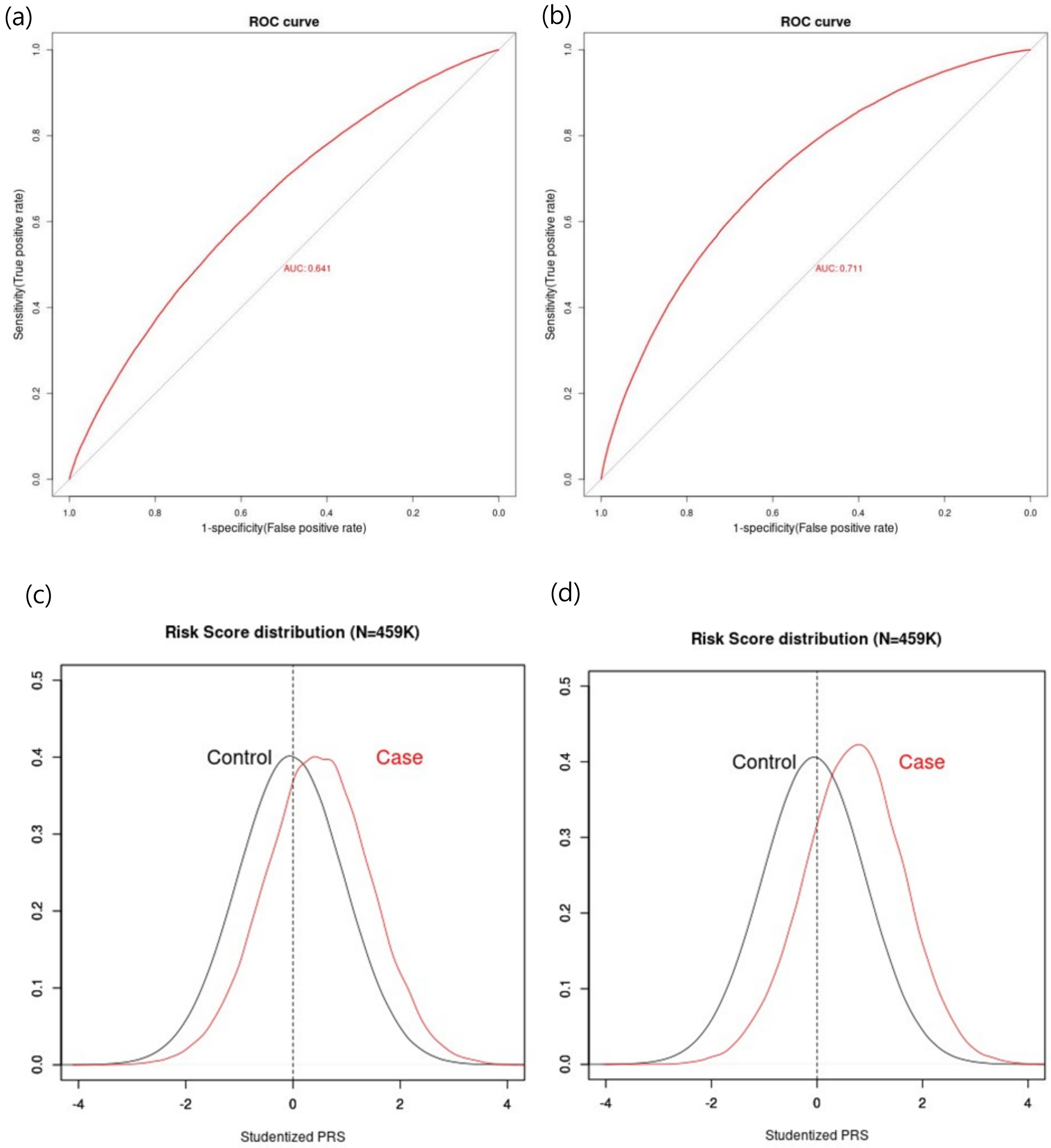

Supplementary Figure 3. The assessment of PH assumption for covariates included in the Survival-All model by performing Schoenfeld residual test.

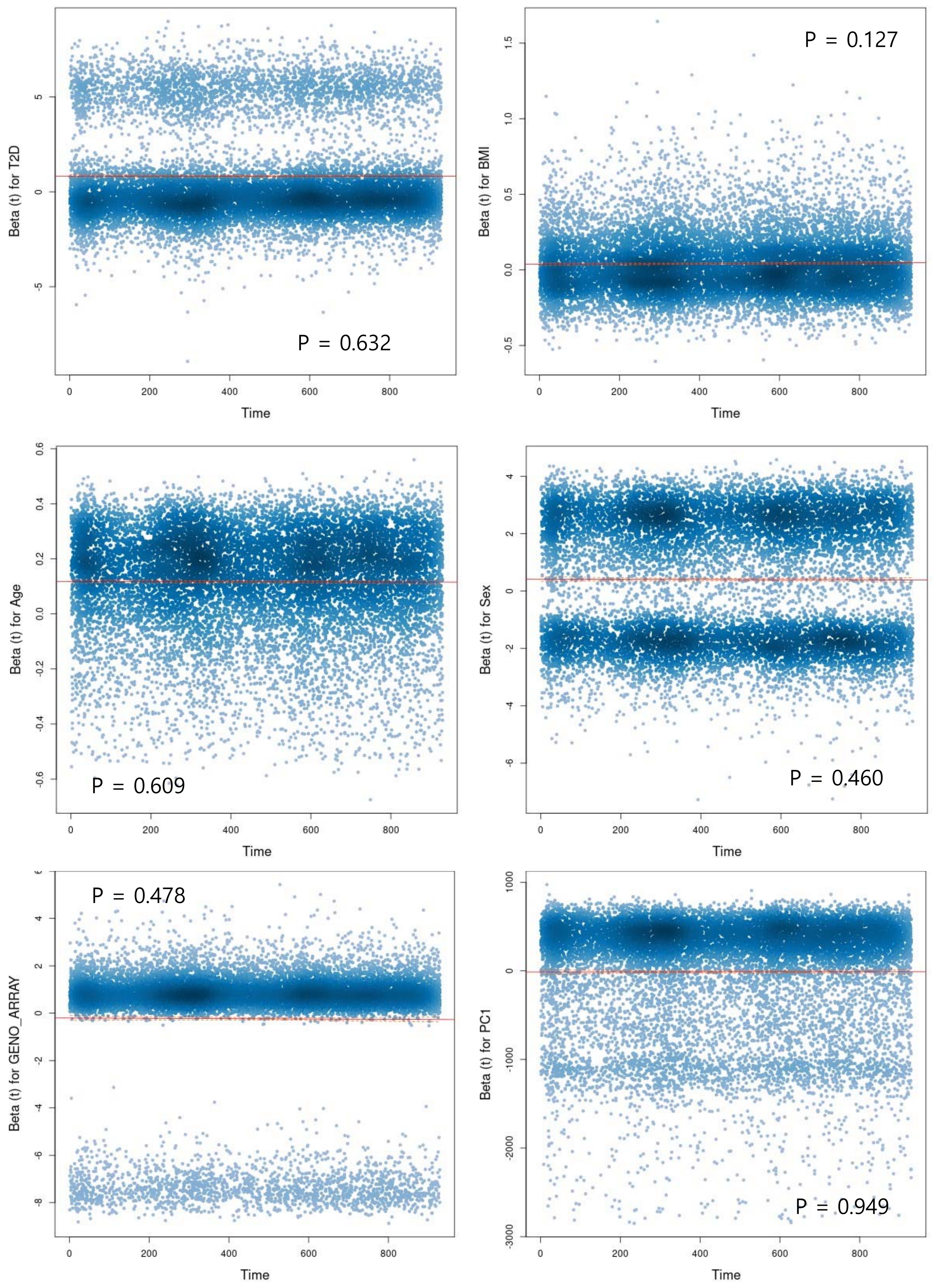

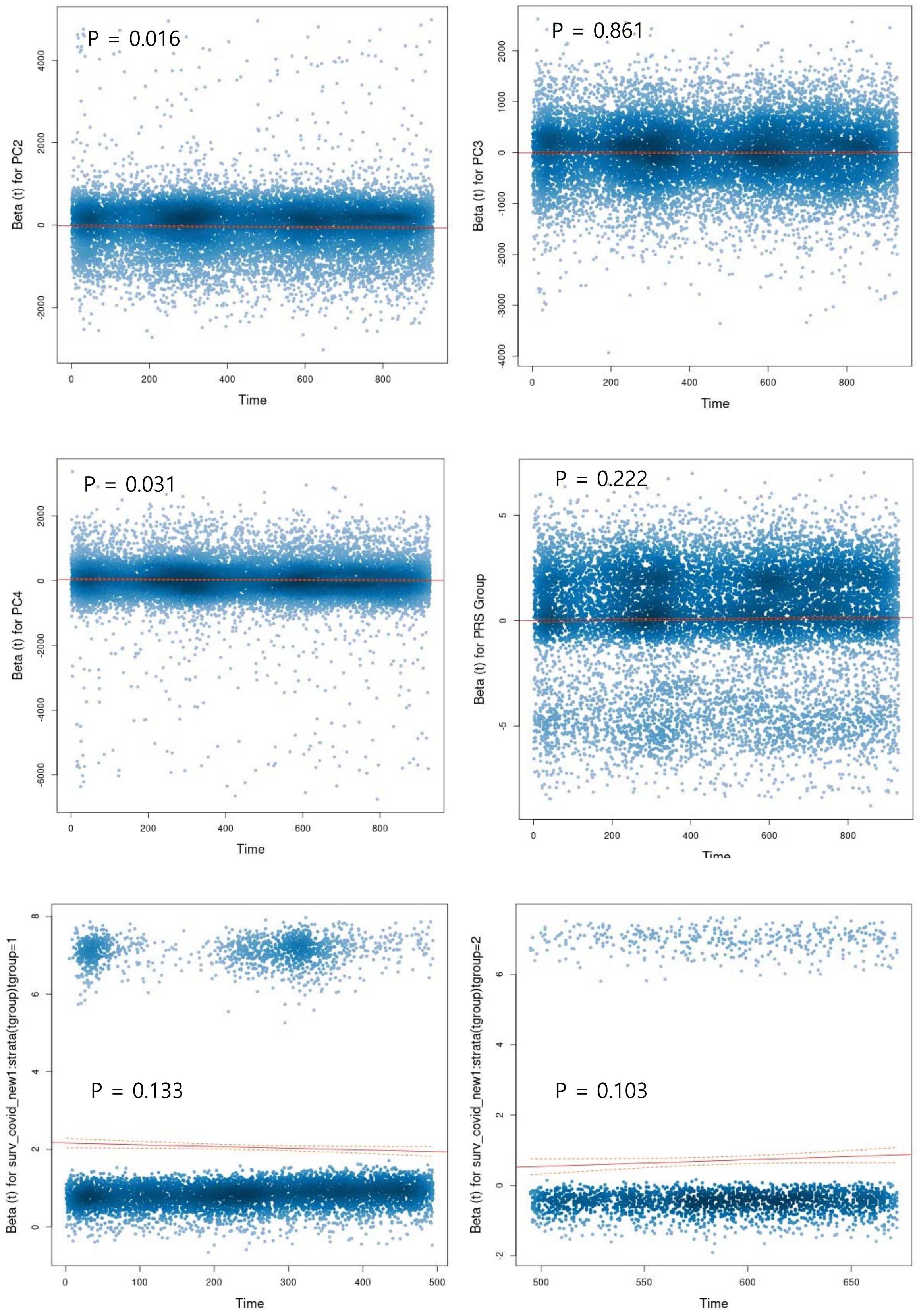
P = 0.016
Beta (t) for PC2
Time
P = 0.861
Beta (t) for PC3
Time
P = 0.031
Beta (t) for PC4
Time
P = 0.222
Beta (t) for PRS Group
Time
P = 0.133
Beta (t) for surv_covid_new1:strata(tgroup)tgroup=1
Time
P = 0.103
Beta (t) for surv_covid_new1:strata(tgroup)tgroup=2
Time

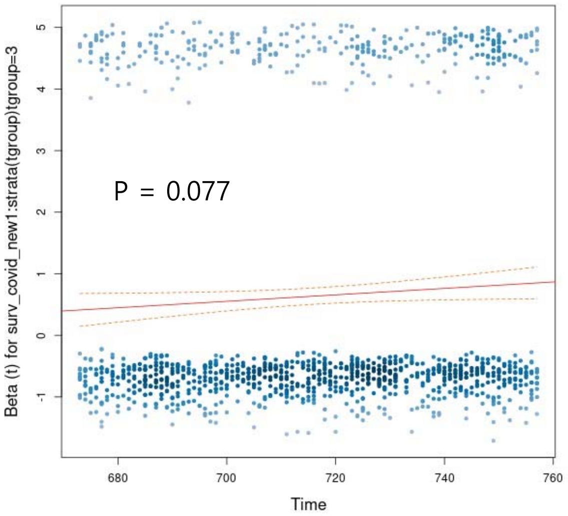
Beta (t) for surv_covid_new1:strata(tgroup)tgroup=3
P = 0.077
Time

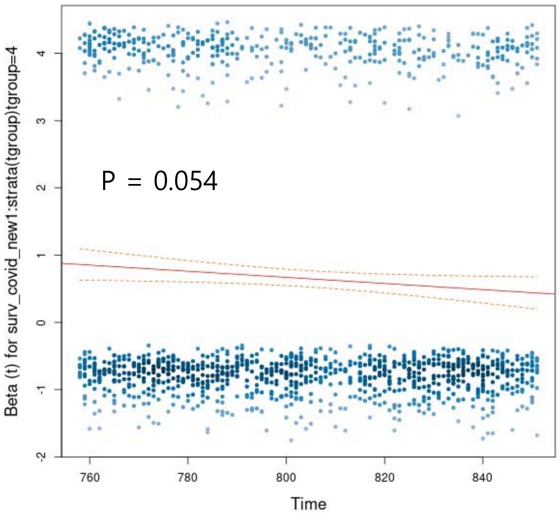
Beta (t) for surv_covid_new1:strata(tgroup)tgroup=4
P = 0.054
Time

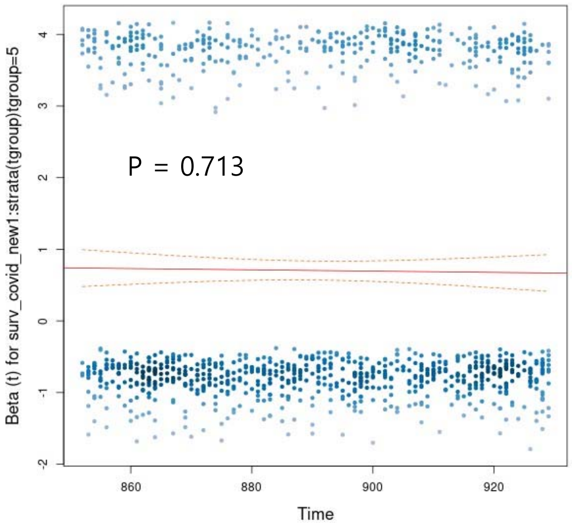
Beta (t) for surv_covid_new1:strata(tgroup)tgroup=5
P = 0.713
Time

Supplementary Figure 4. The Log-Log plot of covariates included in the Survival-All model based on the Kaplan-Meier curves.

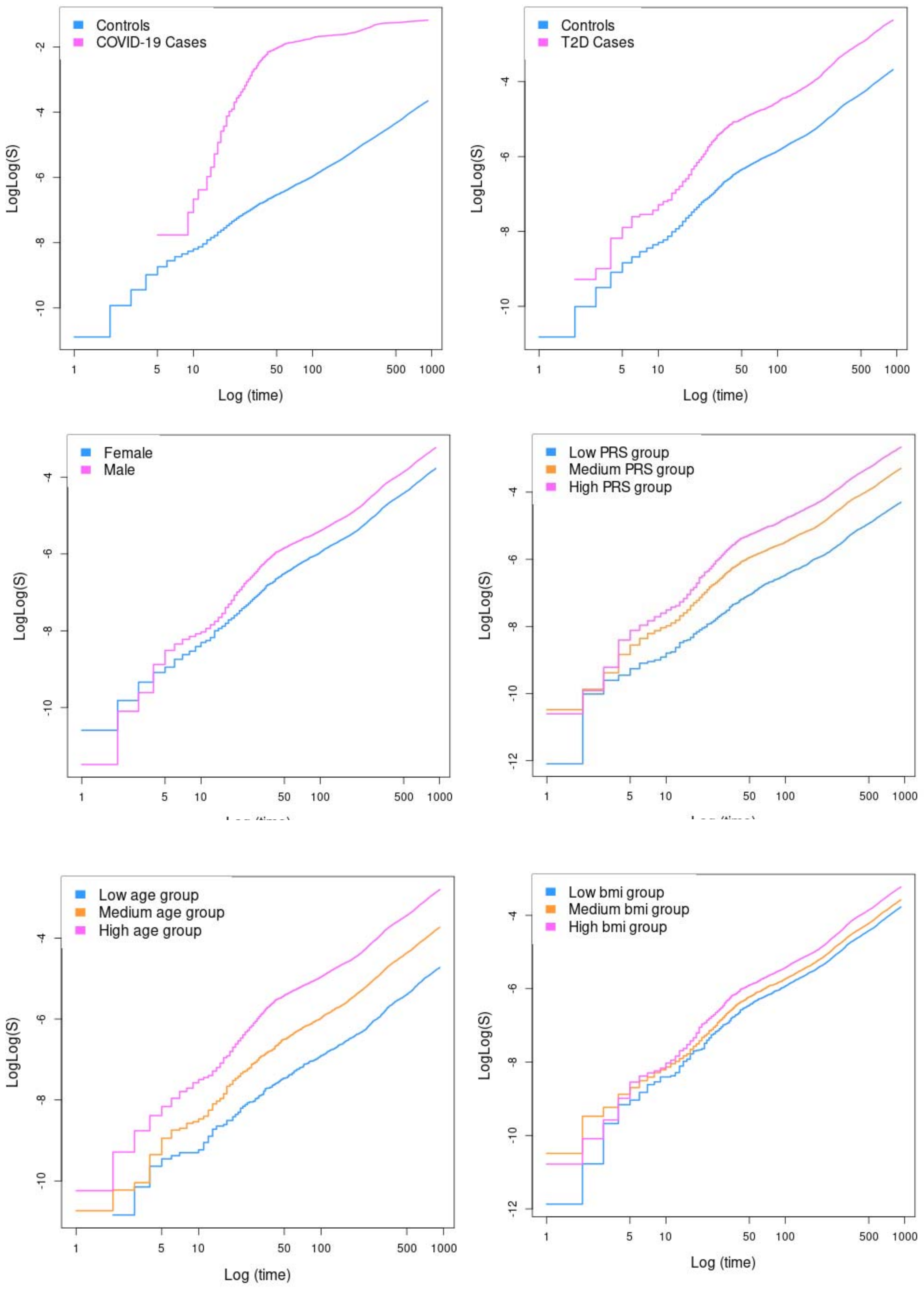

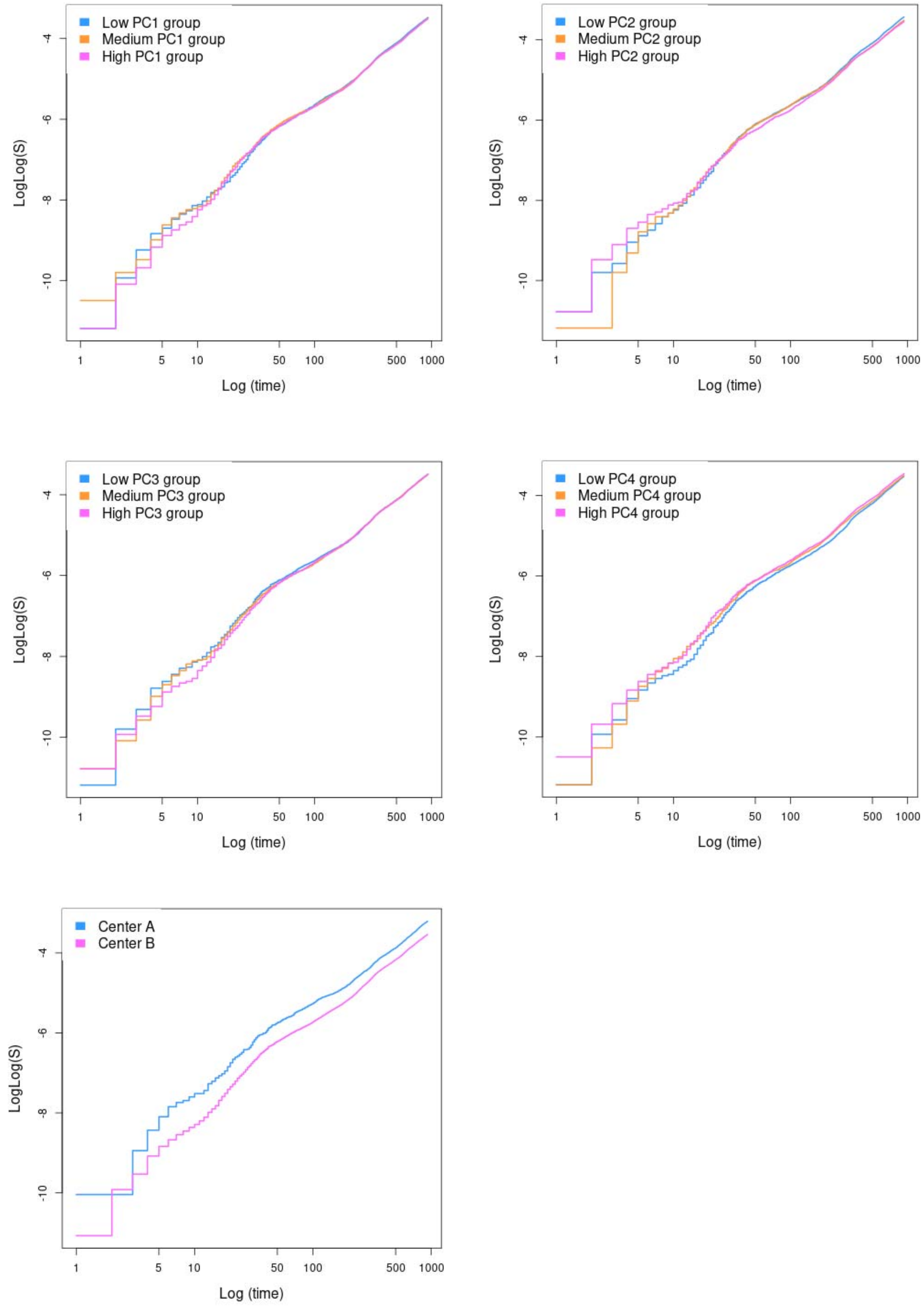
Low PC1 group
Medium PC1 group
High PC1 group
Low PC2 group
Medium PC2 group
High PC2 group
Low PC3 group
Medium PC3 group
High PC3 group
Low PC4 group
Medium PC4 group
High PC4 group
Center A
Center B
LogLog(S)
Log (time)
-4
-6
-8
-10
1
5
10
50
100
500
1000

Supplementary Figure 5. Proportion of COVID-19 variants during the period from 3/11/2020 to 12/20/2022

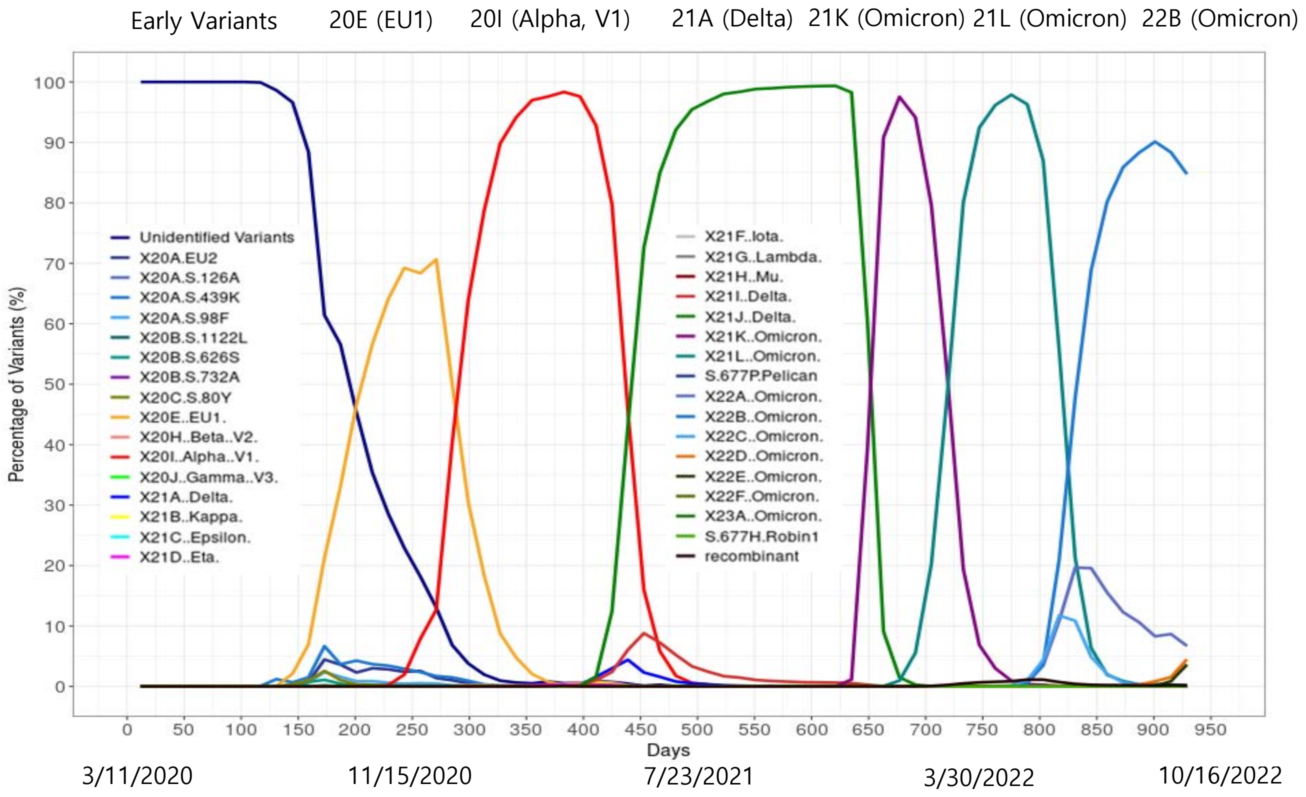

Supplementary Figure 6. The assessment of PH assumption for covariates included in the Survival-COVID-19 model by performing Schoenfeld residual test.

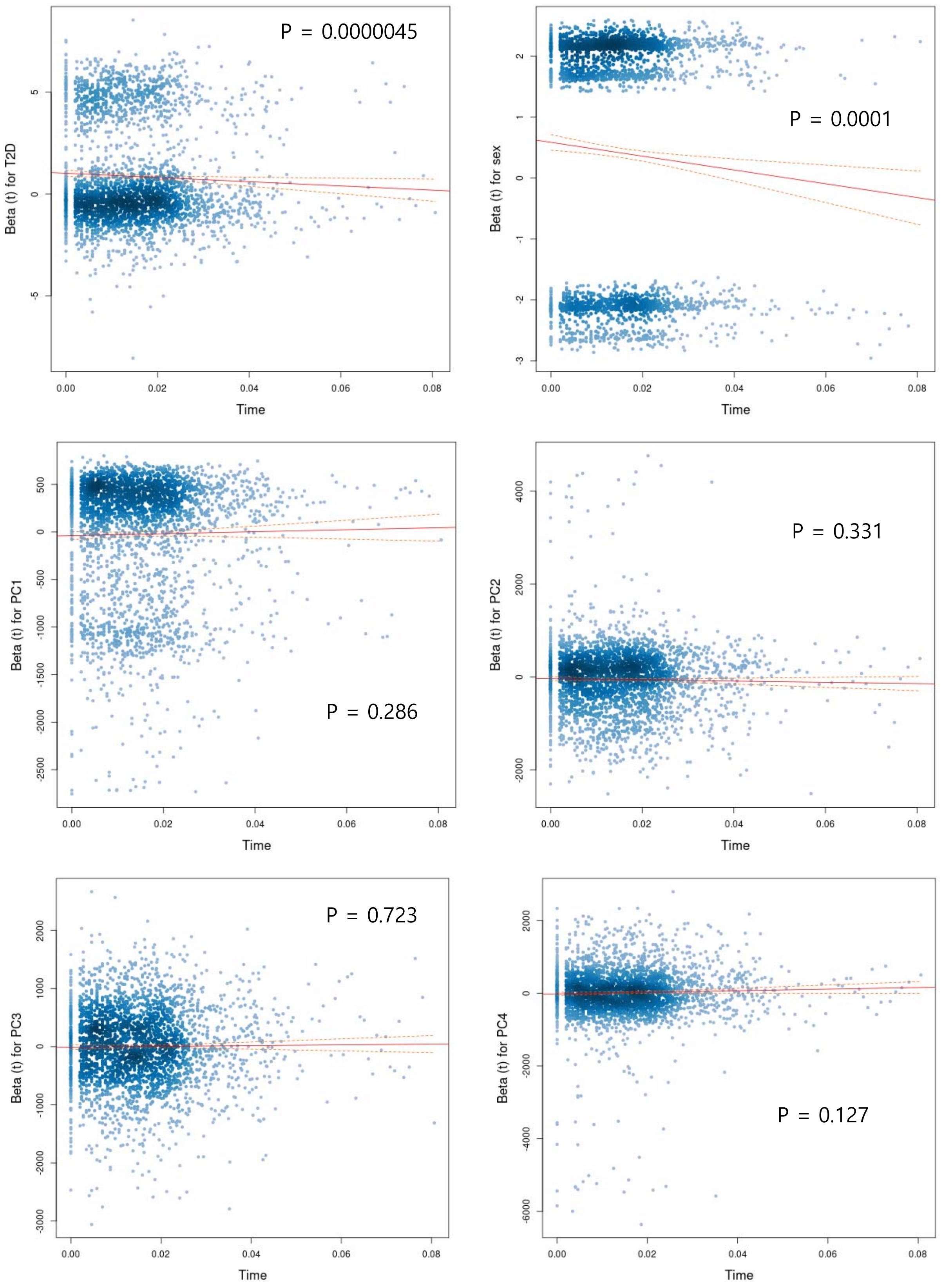

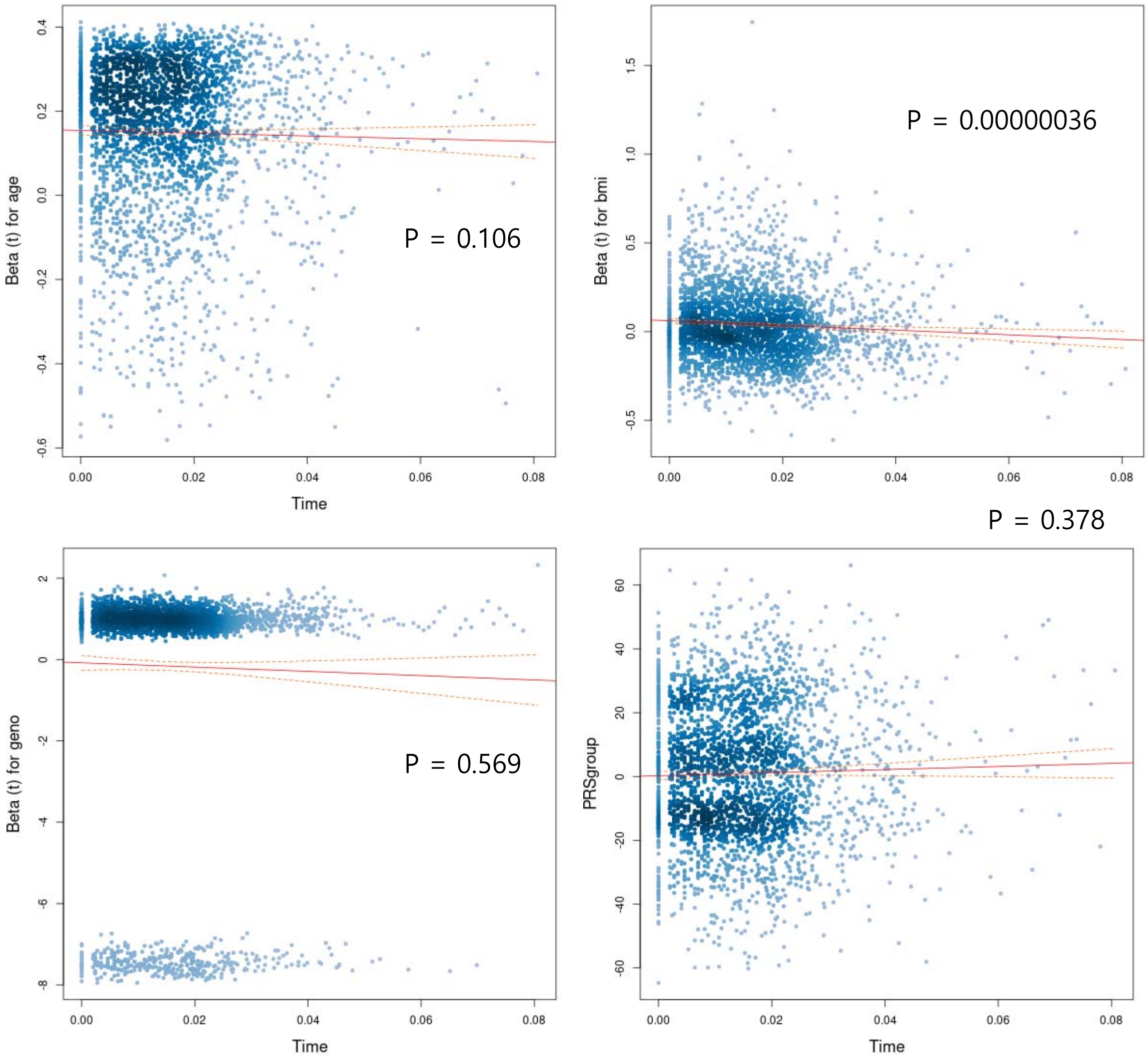
Beta (t) for age
Time
P = 0.106
Beta (t) for bmi
Time
P = 0.00000036
Beta (t) for geno
Time
P = 0.569
PRSgroup
Time
P = 0.378

Supplementary Figure 7. The Log-Log plot of covariates included in the Survival-COVID-19 model based on the Kaplan-Meier curves.

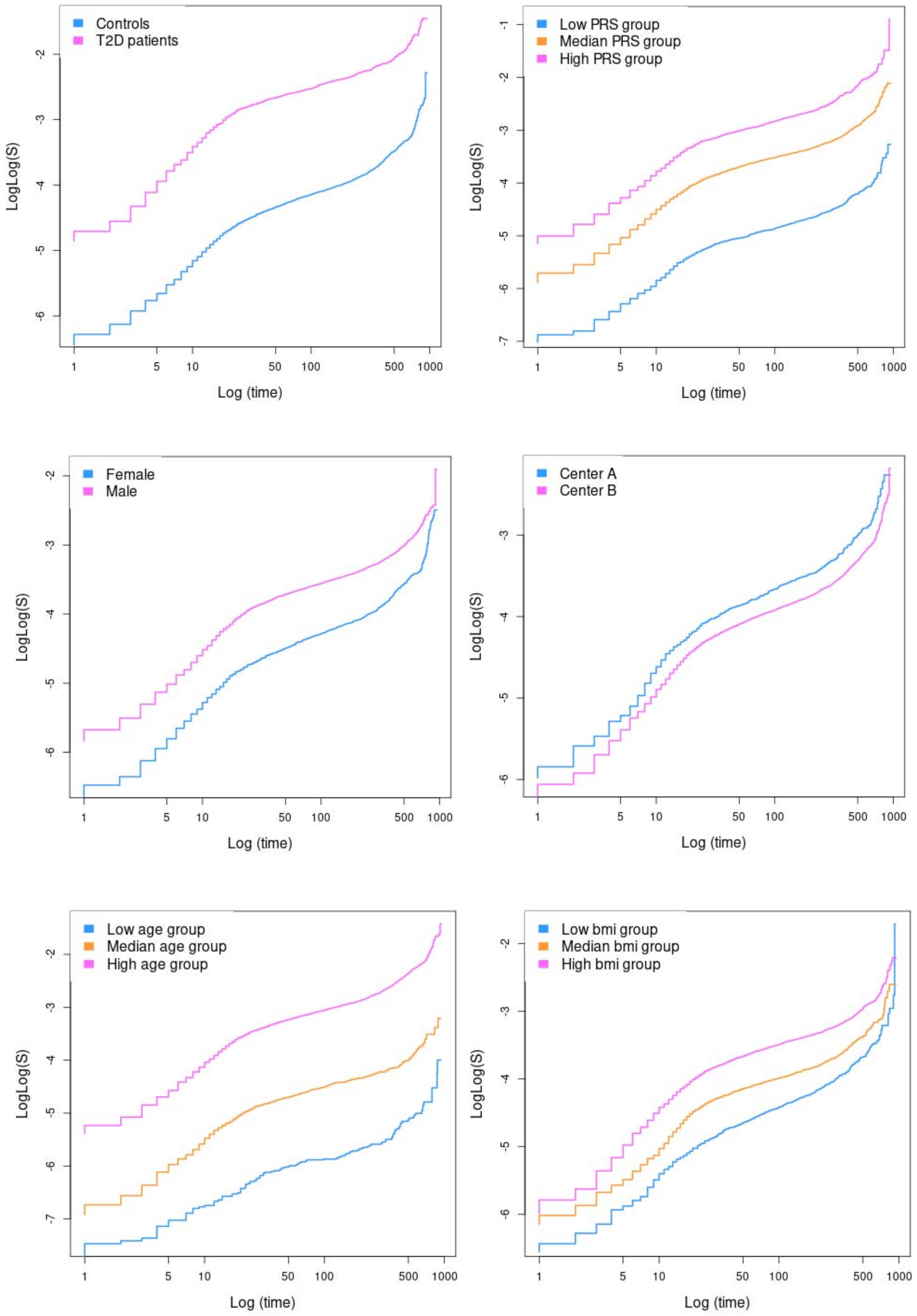

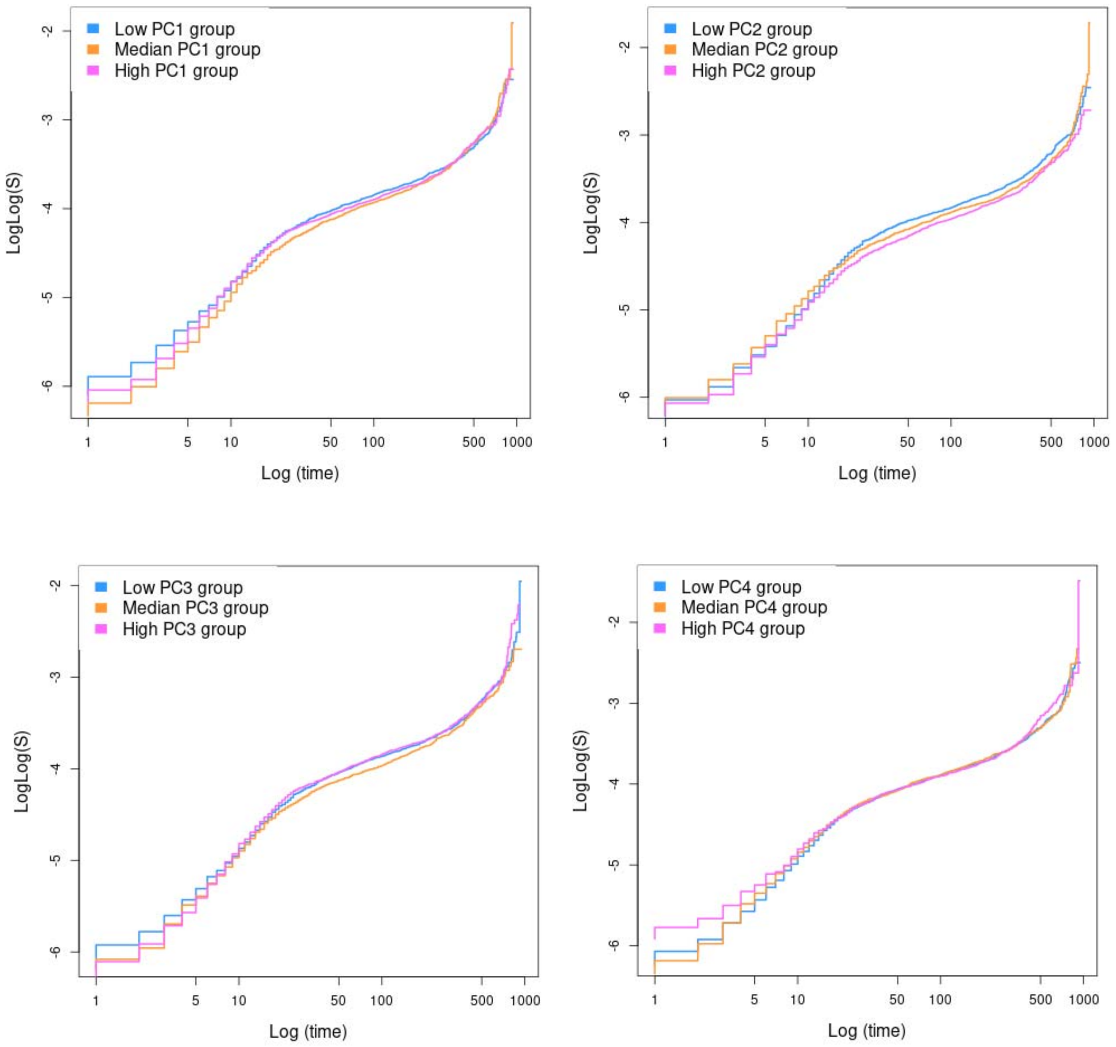
Low PC1 group
Median PC1 group
High PC1 group
LogLog(S)
Log (time)
Low PC2 group
Median PC2 group
High PC2 group
LogLog(S)
Log (time)
Low PC3 group
Median PC3 group
High PC3 group
LogLog(S)
Log (time)
Low PC4 group
Median PC4 group
High PC4 group
LogLog(S)
Log (time)

Supplementary Figure 8. The scatter plot of PRS(T2D) between UK Biobank derived PRS and DIAGRAM derived PRS.

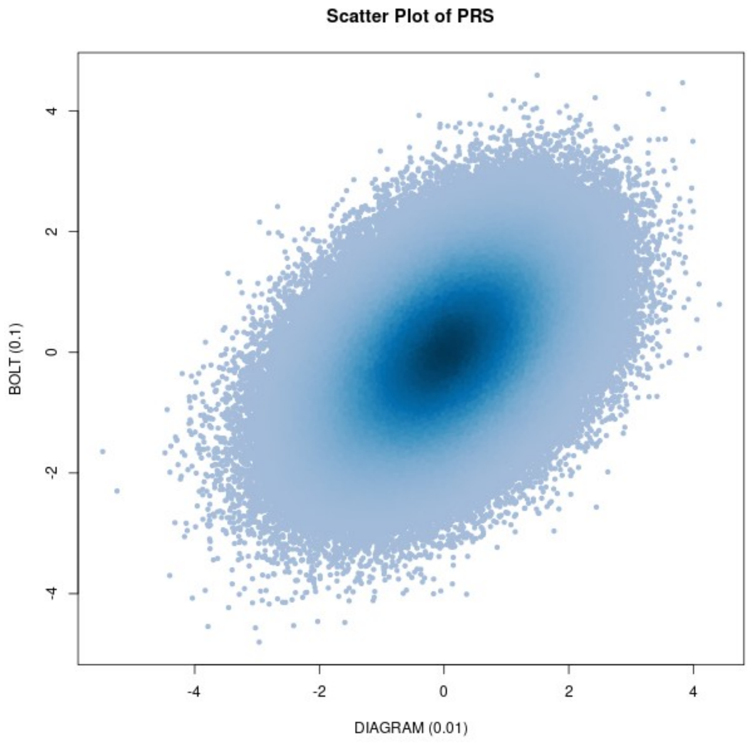

Supplementary Figure 9. The survival curves stratified with PRS groups for T2D derived from the T2D GWAS data from DIAGRAM, and SARS-CoV-2 infection based on the Survival-All model adjusted for the clinical variables (e.g. age, gender, genotyping array and PC 1-4) (left), and the survival curves stratified with PRS groups for T2D based on the Survival-COVID-19 model adjusted for the clinical covariates (right).

(a) (b)

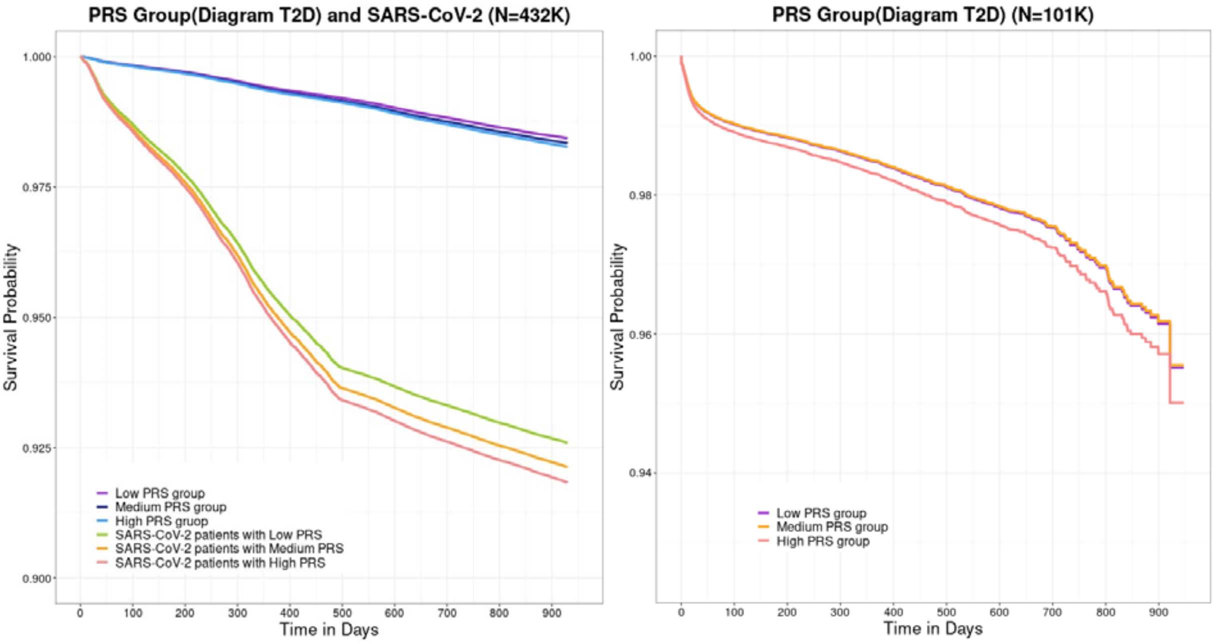

Supplementary Tables

Supplementary Table 1. Estimated heritability and genetic correlations between various traits including T2D, SARS-CoV-2 infection and severe COVID-19.

	**T2D**	**BMI**	**WHR**	**CHD**	**CVD**	**Stroke**	**DBP**	**SBP**	**Asthma**	**SARS-CoV-2**	**COVID-19**
**T2D**	**0.1536 (0.012)**	0.0773 (0.0242)	0.374 (0.0243)	0.375 (0.033)	0.367 (0.0327)	0.2886 (0.0637)	0.128 (0.0255)	0.2162 (0.0223)	0.1298 (0.0273)	-0.0758 (0.0485)	0.2416 (0.0858)
**BMI**	0.0773 (0.0242)	**0.2114 (0.0066)**	0.6043 (0.0176)	0.3811 (0.0254)	0.3992 (0.0232)	0.4189 (0.0576)	0.2749 (0.0174)	0.1388 (0.0164)	0.1833 (0.0219)	0.0571 (0.0317)	0.6379 (0.1117)
**WHR**	0.374 (0.0243)	0.6043 (0.0176)	**0.1405 (0.0049)**	0.4116 (0.023)	0.4312 (0.0223)	0.421 (0.0536)	0.2094 (0.0196)	0.134 (0.0167)	0.1685 (0.0231)	0.0333 (0.0338)	0.6019 (0.1125)
**CHD**	0.375 (0.033)	0.3811 (0.0254)	0.4116 (0.023)	**0.0309 (0.0022)**	0.9905 (0.0041)	0.67 (0.084)	0.2878 (0.0303)	0.3439 (0.0254)	0.1782 (0.0345)	0.0457 (0.0501)	0.7352 (0.1410)
**CVD**	0.367 (0.0327)	0.3992 (0.0232)	0.4312 (0.0223)	0.9905 (0.0041)	**0.03 (0.0019)**	0.766 (0.0616)	0.3218 (0.0289)	0.3493 (0.025)	0.2016 (0.0332)	0.0088 (0.1693)	0.8138 (0.1534)
**Stroke**	0.2886 (0.0637)	0.4189 (0.0576)	0.421 (0.0536)	0.67 (0.084)	0.766 (0.0616)	**0.0057 (0.0009)**	0.4005 (0.058)	0.3056 (0.0522)	0.2665 (0.0677)	-0.2639 (0.0935)	0.8768 (0.2477)
**DBP**	0.128 (0.0255)	0.2749 (0.0174)	0.2094 (0.0196)	0.2878 (0.0303)	0.3218 (0.0289)	0.4005 (0.058)	**0.1059 (0.0041)**	0.6535 (0.017)	0.0532 (0.0208)	-0.1116 (0.0369)	0.1647 (0.0711)
**SBP**	0.2162 (0.0223)	0.1388 (0.0164)	0.134 (0.0167)	0.3439 (0.0254)	0.3493 (0.025)	0.3056 (0.0522)	0.6535 (0.017)	**0.1108 (0.0044)**	0.0288 (0.0214)	-0.1560 (0.0366)	0.0470 (0.0696)
**Asthma**	0.1298 (0.0273)	0.1833 (0.0219)	0.1685 (0.0231)	0.1782 (0.0345)	0.2016 (0.0332)	0.2665 (0.0677)	0.0532 (0.0208)	0.0288 (0.0214)	**0.0467 (0.0046)**	0.0781 (0.0433)	0.2809 (0.0978)
**SARS-CoV-2**	-0.0758 (0.0485)	0.0571 (0.0317)	0.0333 (0.0338)	0.0457 (0.0501)	0.0088 (0.1693)	-0.2639 (0.0935)	-0.1116 (0.0369)	-0.1560 (0.0366)	0.0781 (0.0433)	**0.0240 (0.0028)**	0.3099 (0.1335)
**COVID-19**	0.2416 (0.0858)	0.6379 (0.1117)	0.6019 (0.1125)	0.7352 (0.1410)	0.8138 (0.1534)	0.8768 (0.2477)	0.1647 (0.0711)	0.0470 (0.0696)	0.2809 (0.0978)	0.3099 (0.1335)	**0.0269 (0.0093)**

Diagonal elements: the estimated heritability
Non-diagonal elements: the estimated genetic correlation between pair of traits
Abbreviation: SARS-CoV-2 (SARS-CoV-2 infection), COVID-19 (severe COVID-19)

Supplementary Table 2. The AUCs of PRS for T2D, Diagram T2D, SARS-Cov-2 infection, COVID-19 severity, CHD and R2 of PRS for BMI, Hip, Waist, WHR, HDLC.

**Binary Traits (AUC)**	**T2D**	**Diagram T2D**	**SARS-CoV-2**	**COVID-19**	**CHD**
Clinical	0.659	0.659	0.596	0.643	0.7317
PRS	0.641	0.614	0.532	0.518	0.5463
PRS + Clinical	0.711	0.694	0.602	0.644	0.7363
**Continuous Traits (R2)**	**BMI**	**Hip**	**Waist**	**WHR**	**HDLC**
Clinical	0.0196	0.00330	0.2311	0.4387	0.1807
PRS	0.0170	0.00824	0.01343	0.00824	0.00923
PRS + Clinical	0.0365	0.01150	0.24431	0.44669	0.18948

Clinical: age, age2, gender, genotyping array and PC 1-4

Abbreviation: SARS-CoV-2 (SARS-CoV-2 infection), COVID-19 (severe COVID-19)

Supplementary Table 3. The estimated AUC for the candidate PRS with a range of tuning parameter (fraction of causal variants = 1, 0.3, 0.1, 0.03, 0.01, 0.003, 0.001, inf)

	**1**	**0.3**	**0.1**	**0.03**	**0.01**	**0.003**	**0.001**	**INF**
**T2D**	0.625	0.632	**0.641**	0.609	0.565	0.535	0.521	0.625
**BMI**	**0.0170**	0.0165	0.0157	0.0064	0.0016	0.00024	5.99E-05	0.016
**WHR**	**0.0082**	0.0082	0.0081	0.0033	0.00087	0.00011	2.47E-05	0.008
**CHD**	0.546	**0.5463**	0.5463	0.531	0.5175	0.5081	0.5022	0.546
**CVD**	0.5421	0.5422	**0.5423**	0.5286	0.5158	0.5073	0.5014	0.542
**Stroke**	**0.5331**	0.5326	0.5326	0.5224	0.5125	0.5039	0.5022	0.533
**SARS-CoV-2**	**0.525**	0.526	0.528	0.531	**0.532**	0.522	0.508	0.525
**COVID-19**	0.516	0.516	0.516	0.517	0.517	**0.518**	0.518	0.516

PRS with best discriminative capability were highlighted in bold type
Abbreviation: SARS-CoV-2 (SARS-CoV-2 infection), COVID-19 (severe COVID-19)

Supplementary Table 4. The death status for 432K participants stratified with T2D / T2D PRS, SARS-CoV-2 infection

Death	SARS-CoV-2	Control	Total
T2D	767	2,147	2,914
Control	2,069	7,928	9,997
total	2,836	10,075	12,911
Low PRS	585	2,350	2,935
Medium PRS	1,619	5,641	7,260
High PRS	632	2,084	2,716
Total	2,836	10,075	12,911
**Censored**	**SARS-CoV-2**	**Control**	**Total**
T2D	6,692	22,588	29,280
Control	91,558	298,226	389,784
total	98,250	320,814	419,064
Low PRS	51,845	145,387	197,232
Medium PRS	39,102	145,170	184,272
High PRS	7,303	30,257	37,560
Total	98,250	320,814	419,064
**%Death**	**SARS-CoV-2**	**Control**	**Total**
T2D	10.2829	8.6800	9.0514
Control	2.2098	2.5895	2.5006
T2D	2.8055	3.0448	2.9888
Low PRS	1.4966	1.8890	1.7902
Medium PRS	4.2111	4.0585	4.0905
High PRS	8.2373	6.5328	6.8753
Total	2.8055	3.0448	2.9888

Supplementary Table 5. The assessment of survival models' performances measured by concordance index (C-index) using 10-fold cross validation.

Model	Covariates	C-index
Survival-All	SARS-CoV-2 + T2D + Clinical	0.77
Survival-All	SARS-CoV-2 + T2D PRS group + Clinical	0.755
Survival-COVID-19	T2D + Clinical	0.791
Survival-COVID-19	T2D PRS group + Clinical	0.795

Clinical: age, gender, BMI, genotyping array and PC 1-4

Supplementary Table 6. The results of multivariable logistic regression models representing the effects of T2D and T2D PRS on SARS-CoV-2 infection.

Covariates	OR (95% CI)	P-value
T2D	T2D: 0.885 (0.862 - 0.909)	1.10E-19
T2D + Clinical	T2D: 1.006 (0.979 - 1.035)	6.53E-01
T2D PRS	T2D PRS: 0.995 (0.988 - 1.002)	1.24E-01
T2D PRS + Clinical	T2D PRS: 1.008 (0.998 - 1.018)	1.20E-01
T2D PRS Group + Clinical	Medium: 1.006 (0.988 - 1.025)	4.89E-01
	High: 1.020 (0.988 - 1.053)	2.32E-01
T2D + Vaccine + Clinical	T2D: 1.033 (1.004 - 1.062)	2.44E-02
	Vaccine: 1.293 (1.275 - 1.312)	2.20E-269
T2D PRS	T2D PRS: 0.995 (0.988 - 1.002)	1.24E-01
T2D PRS + Vaccine + Clinical	T2D PRS: 1.012 (1.002 - 1.023)	1.77E-02
	Vaccine: 1.293 (1.274 - 1.312)	4.76E-269
T2D PRS Group + Vaccine + Clinical	Medium: 1.009 (0.990 - 1.027)	3.56E-01
	High: 1.032 (1.000 - 1.066)	5.29E-02
	Vaccine: 1.293 (1.274 - 1.311)	7.46E-269

Clinical: age, gender, BMI, genotyping array and PC 1-4
T2D PRS: susceptibility for T2D adjusted for age and gender using the logistic regression model
T2D PRS Group: low, medium and High T2D PRS groups

Supplementary Table 7. Association of various disease traits with SARS-CoV-2 infection using multivariable logistic regression models adjusted for the potential confounders (N = 408,888).

Models	CHD		CVD	
	OR (95% CI)	P-value	OR (95% CI)	P-value
**Trait**	0.763 (0.742 - 0.785)	2.66E-78	0.746 (0.727 - 0.765)	1.08E-109
**Trait + Clinical**	0.957 (0.929 - 0.985)	2.99E-03	0.928 (0.904 - 0.953)	4.37E-08
**PRS**	1.009 (0.953 - 1.069)	7.48E-01	1.014 (0.956 - 1.075)	6.49E-01
**PRS + Clinical**	0.987 (0.931 - 1.046)	6.61E-01	0.991 (0.935 - 1.052)	7.77E-01
**Models**	**Stroke**		**BMI**	
	OR (95% CI)	P-value	OR (95% CI)	P-value
**Trait**	0.690 (0.656 - 0.725)	6.95E-48	1.005 (0.998 - 1.013)	1.54E-01
**Trait + Clinical**	0.833 (0.792 - 0.876)	1.59E-12	1.030 (1.023 - 1.038)	6.90E-15
**PRS**	1.014 (0.956 - 1.075)	6.49E-01	1.013 (0.955 - 1.074)	6.69E-01
**PRS + Clinical**	0.991 (0.935 - 1.052)	7.76E-01	0.990 (0.934 - 1.051)	7.51E-01
**Models**	**Hip**		**Waist**	
	OR (95% CI)	P-value	OR (95% CI)	P-value
**Trait**	1.038 (1.031 - 1.046)	2.70E-23	0.993 (0.986 - 1.000)	6.03E-02
**Trait + Clinical**	1.048 (1.040 - 1.056)	3.23E-34	1.045 (1.037 - 1.054)	1.08E-24
**PRS**	1.014 (0.957 - 1.075)	6.34E-01	1.014 (0.957 - 1.075)	6.33E-01
**PRS + Clinical**	0.992 (0.935 - 1.053)	7.94E-01	0.992 (0.935 - 1.053)	7.95E-01
**Models**	**WHR**		**HDLC**	
	OR (95% CI)	P-value	OR (95% CI)	P-value
**Trait**	0.960 (0.953 - 0.968)	1.03E-26	0.987 (0.979 - 0.995)	1.37E-03
**Trait + Clinical**	1.024 (1.014 - 1.035)	2.38E-06	0.998 (0.989 - 1.007)	6.02E-01
**PRS**	1.015 (0.957 - 1.076)	6.26E-01	1.001 (0.940 - 1.065)	9.76E-01
**PRS + Clinical**	0.993 (0.936 - 1.053)	8.03E-01	0.978 (0.918 - 1.041)	4.82E-01

Trait: CHD, CVD, Stroke, BMI, Hip circumference, Waist circumference, WHR, HDLC
Clinical: age, gender, genotyping array and PC 1-4

Supplementary Table 8. SNP lists and weights for cluster-specific partitioned polygenic scores from DiCorpo et al (2022).

Cluster	SNP	Locus	Allele	Weight
BetaCell	rs10830961	MTNR1B	G	3.806
	rs7756992	CDKAL1	G	3.049
	rs4502156	C2CD4A	T	2.682
	rs1111875	HHEX	C	2.656
	rs7903146	TCF7L2	T	2.468
	rs3802177	SLC30A8	G	2.407
	rs10811661	CDKN2A_B	T	1.766
	rs11257655	CDC123/CAMK1D	T	1.587
	rs7965349	OASL	C	1.555
	rs1371135	AP3S2	T	1.542
	rs17265513	ZHX3	C	1.369
	rs1496653	UBE2E2	A	1.350
	rs1996546	ACSL1	G	1.349
	rs12899811	PRC1	G	1.262
	rs8108269	GIPR	G	1.255
	rs17676067	GLP2R	C	1.216
	rs5215	KCNJ11	C	1.191
	rs233449	KCNQ1	G	1.184
	rs505922	ABO	C	1.169
	rs516946	ANK1	C	1.121
	rs10758593	GLIS3	A	1.094
	rs4430796	HNF1B	G	1.038
	rs9921586	CTRB2	G	1.005
	rs2184061	CDKN2A	A	0.971
	rs2334499	DUSP8	T	0.959
	rs11708067	ADCY5	A	0.919
	rs15563	GIP	G	0.883
	rs4812829	HNF4A	A	0.844
	rs3736505	HSD17B12	G	0.781
	rs17791513	TLE4	A	0.776
Proinsulin	rs1552224	ARAP1	A	4.484
	rs1359790	SPRY2	G	1.073
	rs10276674	DGKB	C	1.001
	rs4402960	IGF2BP2	T	0.969
	rs11063069	CCND2	G	0.812
	rs4812829	HNF4A	A	0.793

	rs11257655	CDC123/CAMK1D	T	0.780
Obesity	rs11075990	FTO	G	4.963
	rs12970134	MC4R	A	2.783
	rs10146997	NRXN3	G	1.715
	rs3736505	HSD17B12	G	1.240
	rs6742799	RBMS1	A	1.036
Lipodystrophy	rs2943641	IRS1	C	3.008
	rs13389219	GRB14	C	2.738
	rs2881654	PPARG	G	2.107
	rs2820443	LYPLAL1	T	1.919
	rs459193	ANKRD55	G	1.799
	rs2925979	CMIP	T	1.744
	rs3996352	KLF14	A	1.628
	rs10503669	LPL	C	1.536
	rs3843467	ANKRD55	T	1.309
	rs702634	ARL15	A	1.286
	rs11708067	ADCY5	A	1.242
	rs9891146	C17orf58	T	1.078
	rs3132524	POU5F1	C	1.056
	rs2296172	MACF1	G	1.031
	rs4457053	ZBED3	G	0.964
	rs2276853	KIF9	A	0.956
	rs6795735	ADAMTS9	C	0.949
	rs11063069	CCND2	G	0.938
	rs17106184	FAF1	G	0.812
	rs1106240	MPHOSPH9	T	0.786
Liver/Lipid	rs780094	GCKR	C	5.340
	rs16996148	CILP2	T	2.054
	rs9271775	HLA-DQA1	T	0.976
	rs738408	PNPLA3	T	0.841
	rs7961581	TSPAN8/LGR5	C	0.832

Supplementary Table 9. The AUCs of PRS and number of SNPs for T2D cluster

	**Beta Cell**	**Proinsulin**	**Obesity**	**Lipodystrophy**	**Liver/Lipid**
#SNPs	30	7	5	20	5
AUC of PRS	0.566	0.528	0.523	0.543	0.517
AUC of adjusted PRS	0.671	0.661	0.660	0.664	0.660

Adjusted PRS: PRS adjusted for age, gender, BMI, genotyping array, and the top 4 PCs using the logistic regression model

Supplementary Table 10. The results of proportional odds models for the association of COVID-19 severity and T2D cluster

Cluster	Covariates	OR (95% CI)	P-value	Brant test
Beta Cell	PRS + Clinical	PRS: 1.03 (0.986 - 1.08)	1.74E-01	0.16
Proinsulin	PRS + Clinical	PRS: 1.07 (0.991 - 1.156)	8.52E-02	0.02
Obesity	PRS + Clinical	PRS: 1.14 (1.05 - 1.237)	1.70E-03	0.07
Lipodystrophy	PRS + Clinical	PRS: 1.09 (1.025 - 1.158)	5.56E-03	0.01
Liver/Lipid	PRS + Clinical	PRS: 1.16 (1.065 - 1.276)	8.65E-04	0.01

Supplementary Table 11. The results of Cox PH model with all participants to identify the effects of T2D cluster and SARS-CoV-2 infection on mortality for 432K participants from UK Biobank

Cluster	Covariates	HR (95% CI)	P-value
Beta Cell	PRS + Clinical	PRS: 1.013 (0.982 - 1.045)	4.01E-01
	PRS + SARS-CoV-2 + Clinical	PRS: 1.009 (0.978 - 1.040)	5.77E-01
		tgroup1: 7.763 (7.310 - 8.243)	2.23E-308
		tgroup2: 2.057 (1.850 - 2.286)	1.25E-40
		tgroup3: 1.882 (1.652 - 2.144)	1.85E-21
		tgroup4: 1.923 (1.708 - 2.166)	3.45E-27
		tgroup5: 2.006 (1.761 - 2.284)	9.94E-26
Proinsulin	PRS + Clinical	PRS: 0.973 (0.917 - 1.033)	3.78E-01
	PRS + SARS-CoV-2 + Clinical	PRS: 0.955 (0.900 - 1.013)	1.24E-01
		tgroup1: 7.768 (7.316 - 8.249)	2.23E-308
		tgroup2: 2.057 (1.851 - 2.287)	1.12E-40
		tgroup3: 1.883 (1.653 - 2.146)	1.69E-21
		tgroup4: 1.924 (1.709 - 2.167)	3.15E-27
		tgroup5: 2.007 (1.762 - 2.286)	9.22E-26
Obesity	PRS + Clinical	PRS: 1.026 (0.961 - 1.095)	4.50E-01
	PRS + SARS-CoV-2 + Clinical	PRS: 1.000 (0.938 - 1.067)	1.00E+00
		tgroup1: 7.763 (7.311 - 8.244)	2.23E-308
		tgroup2: 2.057 (1.850 - 2.286)	1.22E-40
		tgroup3: 1.883 (1.653 - 2.145)	1.82E-21
		tgroup4: 1.924 (1.708 - 2.166)	3.40E-27
		tgroup5: 2.006 (1.761 - 2.285)	9.84E-26
Lipodystrophy	PRS + Clinical	PRS: 1.033 (0.989 - 1.079)	1.44E-01
	PRS + SARS-CoV-2 + Clinical	PRS: 1.022 (0.9785 - 1.067)	3.28E-01
		tgroup1: 7.761 (7.308 - 8.241)	2.23E-308
		tgroup2: 2.056 (1.85 - 2.286)	1.30E-40
		tgroup3: 1.882 (1.652 - 2.144)	1.87E-21
		tgroup4: 1.923 (1.708 - 2.166)	3.51E-27
		tgroup5: 2.006 (1.761 - 2.284)	1.01E-25
Liver/Lipid	PRS + Clinical	PRS: 0.985 (0.913- 1.064)	7.04E-01
	PRS + SARS-CoV-2 + Clinical	PRS: 0.966 (0.896 - 1.042)	3.73E-01
		tgroup1: 7.766 (7.313 - 8.246)	2.23E-308
		tgroup2: 2.057 (1.85 - 2.287)	1.20E-40
		tgroup3: 1.883 (1.653 - 2.145)	1.76E-21
		tgroup4: 1.924 (1.709 - 2.166)	3.28E-27
		tgroup5: 2.006 (1.762 - 2.285)	9.54E-26

Supplementary Table 12. The results of Cox PH model with SARS-CoV-2 infected individuals to identify the effect of T2D cluster on survival times only for 101K COVID-19 confirmed cases.

Cluster	Covariates	HR (95% CI)	P-value
Beta Cell	PRS + Clinical	PRS: 1.033 (0.969 - 1.101)	3.22E-01
	PRS + SARS-CoV-2 variants + Clinical	PRS: 1.014 (0.952 - 1.08)	6.69E-01
		Early: 8.33 (6.916 - 10.032)	1.38E-110
		EU1: 4.602 (3.786 - 5.593)	4.11E-53
		Alpha: 5.501 (4.531 - 6.679)	1.76E-66
		Delta: 1.351 (1.111 - 1.644)	2.64E-03
		Omicron1: 0.838 (0.682 - 1.029)	0.0915
		Omicron2: 0.781 (0.637 - 0.957)	0.0174
Proinsulin	PRS + Clinical	PRS: 0.934 (0.825 - 1.057)	2.79E-01
	PRS + SARS-CoV-2 variants + Clinical	PRS: 0.903 (0.8 - 1.021)	1.03E-01
		Early: 8.345 (6.929 - 10.05)	8.65E-111
		EU1: 4.607 (3.791 - 5.599)	3.46E-53
		Alpha: 5.5 (4.53 - 6.677)	1.78E-66
		Delta: 1.351 (1.11 - 1.644)	2.66E-03
		Omicron1: 0.838 (0.682 - 1.029)	9.14E-02
		Omicron2: 0.781 (0.637 - 0.958)	1.75E-02
Obesity	PRS + Clinical	PRS: 1.02 (0.891 - 1.167)	7.77E-01
	PRS + SARS-CoV-2 variants + Clinical	PRS: 0.958 (0.839 - 1.093)	5.22E-01
		Early: 8.338 (6.923 - 10.042)	1.04E-110
		EU1: 4.602 (3.787 - 5.593)	4.04E-53
		Alpha: 5.504 (4.533 - 6.682)	1.57E-66
		Delta: 1.351 (1.11 - 1.644)	2.65E-03
		Omicron1: 0.837 (0.682 - 1.029)	9.11E-02
		Omicron2: 0.781 (0.637 - 0.957)	1.74E-02
Lipodystrophy	PRS + Clinical	PRS: 1.013 (0.925 - 1.109)	7.87E-01
	PRS + SARS-CoV-2 variants + Clinical	PRS: 0.987 (0.902 - 1.08)	7.73E-01
		Early: 8.337 (6.922 - 10.04)	1.09E-110
		EU1: 4.602 (3.786 - 5.593)	4.11E-53
		Alpha: 5.504 (4.534 - 6.683)	1.54E-66
		Delta: 1.352 (1.111 - 1.645)	2.61E-03
		Omicron1: 0.838 (0.682 - 1.029)	9.19E-02
		Omicron2: 0.781 (0.637 - 0.958)	1.75E-02
Liver/Lipid	PRS + Clinical	PRS: 0.859 (0.73 - 1.011)	6.68E-02
	PRS + SARS-CoV-2 variants + Clinical	PRS: 0.828 (0.706 - 0.971)	2.00E-02
		Early: 8.345 (6.929 - 10.05)	8.61E-111

	EU1: 4.599 (3.784 - 5.59)	4.50E-53
	Alpha: 5.502 (4.532 - 6.68)	1.63E-66
	Delta: 1.35 (1.109 - 1.642)	2.75E-03
	Omicron1: 0.837 (0.681 - 1.028)	9.00E-02
	Omicron2: 0.781 (0.637 - 0.958)	1.74E-02
