# Supplementary material for: Type 2 diabetes and its genetic susceptibility are associated with increased severity and mortality of COVID-19 in UK Biobank
Source: Commun Biol. 2024 Jan 24;7:122. doi: 10.1038/s42003-024-05799-1 (PMC10808197; doi:10.1038/s42003-024-05799-1)
Supplement: Supplementary file 3 — Reporting Summary [file 42003_2024_5799_MOESM3_ESM.pdf]

nature portfolio

Corresponding author(s): Dr. Wonil Chung

Last updated by author(s): Dec 31, 2023

Reporting Summary

Nature Portfolio wishes to improve the reproducibility of the work that we publish. This form provides structure for consistency and transparency in reporting. For further information on Nature Portfolio policies, see our Editorial Policies and the Editorial Policy Checklist.

Statistics

For all statistical analyses, confirm that the following items are present in the figure legend, table legend, main text, or Methods section.

n/a	Confirmed	
☐	☒	The exact sample size (*n*) for each experimental group/condition, given as a discrete number and unit of measurement
☐	☒	A statement on whether measurements were taken from distinct samples or whether the same sample was measured repeatedly
☐	☒	The statistical test(s) used AND whether they are one- or two-sided<br>*Only common tests should be described solely by name; describe more complex techniques in the Methods section.*
☐	☒	A description of all covariates tested
☐	☒	A description of any assumptions or corrections, such as tests of normality and adjustment for multiple comparisons
☐	☒	A full description of the statistical parameters including central tendency (e.g. means) or other basic estimates (e.g. regression coefficient) AND variation (e.g. standard deviation) or associated estimates of uncertainty (e.g. confidence intervals)
☐	☒	For null hypothesis testing, the test statistic (e.g. *F*, *t*, *r*) with confidence intervals, effect sizes, degrees of freedom and *P* value noted<br>*Give P values as exact values whenever suitable.*
☐	☒	For Bayesian analysis, information on the choice of priors and Markov chain Monte Carlo settings
☐	☒	For hierarchical and complex designs, identification of the appropriate level for tests and full reporting of outcomes
☐	☒	Estimates of effect sizes (e.g. Cohen's *d*, Pearson's *r*), indicating how they were calculated

Our web collection on statistics for biologists contains articles on many of the points above.

Software and code

Policy information about availability of computer code

Data collection	No software was used to collect the data. Instead, we utilized data from the UK Biobank cohort (application numbers 45052, 58105, 77890) for our analyses.
Data analysis	We utilized BOLT-LMM v2.3 software to conduct GWAS for various phenotypes in the UK Biobank and used LDSC software to estimate heritability and genetic correlations between T2D, its related traits, and COVID-19. We used LDpred software to obtain PRS scores for our traits of interest. Statistical analyses were performed using R (version 4.2.0).

For manuscripts utilizing custom algorithms or software that are central to the research but not yet described in published literature, software must be made available to editors and reviewers. We strongly encourage code deposition in a community repository (e.g. GitHub). See the Nature Portfolio guidelines for submitting code & software for further information.

Data

Policy information about availability of data

All manuscripts must include a data availability statement. This statement should provide the following information, where applicable:

- Accession codes, unique identifiers, or web links for publicly available datasets
- A description of any restrictions on data availability
- For clinical datasets or third party data, please ensure that the statement adheres to our policy

All data generated in the manuscript are available from the corresponding author upon request.

Research involving human participants, their data, or biological material

Policy information about studies with human participants or human data. See also policy information about sex, gender (identity/presentation), and sexual orientation and race, ethnicity and racism.

Reporting on sex and gender	For our analysis, we utilized questionnaire-based self-reported data, hospital inpatient data, and death data. We also incorporated COVID-19 test result data, which included records of PCR tests for SARS-CoV-2. Of the 459,119 UK Biobank samples, we have 249,227 females (54.28%) and 209,892 males (45.72%) based on self-reported gender information.
Reporting on race, ethnicity, or other socially relevant groupings	We restricted our study to only individuals of European ancestry based on the information of self-reported ethnicity.
Population characteristics	UK Biobank is a prospective cohort study comprising over 500,000 individuals from across the United Kingdom. Participants, aged between 40 and 69, were invited to one of 22 centres across the UK from 2006 to 2010. They provided blood, urine, and saliva samples; underwent physical measurements; and answered an extensive questionnaire centered on health and lifestyle questions.
Recruitment	UK Biobank recruited 500,000 people aged between 40 and 69 years from 2006 to 2010 from across the country to participate in the project. Participants underwent various measures, provided blood, urine, and saliva samples for future analysis, gave detailed information about themselves, and agreed to have their health monitored. Over many years, this will build into a powerful resource to help scientists determine why some individuals develop particular diseases while others do not.
Ethics oversight	The UK Biobank is a major national and international health resource and a registered charity in its own right. Its aim is to improve the prevention, diagnosis, and treatment of a wide range of serious and life-threatening illnesses – including cancer, heart diseases, stroke, diabetes, arthritis, osteoporosis, eye disorders, depression, and various forms of dementia.

Note that full information on the approval of the study protocol must also be provided in the manuscript.

Field-specific reporting

Please select the one below that is the best fit for your research. If you are not sure, read the appropriate sections before making your selection.

☒ Life sciences ☐ Behavioural & social sciences ☐ Ecological, evolutionary & environmental sciences

For a reference copy of the document with all sections, see nature.com/documents/nr-reporting-summary-flat.pdf

Life sciences study design

All studies must disclose on these points even when the disclosure is negative.

Sample size	Of the over 500,000 subjects with their phenotypes in the UK Biobank, 488,377 were genotyped. Individuals of non-European ancestry were excluded, resulting in 459,119 samples. We used the full set of UK Biobank samples for our analysis.
Data exclusions	No data were excluded from our analyses.
Replication	In the sensitivity analysis, we computed individual T2D PRS based on the GWAS result from the DIAGRAM consortium for 459,119 participants. We included three PRS groups based on the DIAGRAM-based T2D PRS as covariates in both the Survival-All and the Survival-COVID-19 models. The overall patterns of the estimated survival curves, stratified by T2D and PRS groups, as well as survival curves stratified by PRS groups are similar to the corresponding curves.
Randomization	For n-fold cross-validation, the data is randomly divided into n equal-sized subsets. Of these, n-1 subsets are used for training, and the remaining subset is used for validation. Additionally, when only a portion of the samples was used for analysis, individuals were randomly selected from the full set of samples.
Blinding	Since we did not allocate any groups for our analyses, blinding is not applicable to our study.

Reporting for specific materials, systems and methods

We require information from authors about some types of materials, experimental systems and methods used in many studies. Here, indicate whether each material, system or method listed is relevant to your study. If you are not sure if a list item applies to your research, read the appropriate section before selecting a response.

Materials & experimental systems

n/a	Involved in the study	
☒	☐	Antibodies
☒	☐	Eukaryotic cell lines
☒	☐	Palaeontology and archaeology
☒	☐	Animals and other organisms
☒	☐	Clinical data
☒	☐	Dual use research of concern
☒	☐	Plants

Methods

n/a	Involved in the study	
☒	☐	ChIP-seq
☒	☐	Flow cytometry
☒	☐	MRI-based neuroimaging
